# High-Temperature Thermal Camouflage Device Considering Radiative Thermal Transfer from the Target

**DOI:** 10.3390/mi16080840

**Published:** 2025-07-22

**Authors:** Zeyu Lin, Xiaohong Wang, Jiangtai Lin, Honghao Jiang, Guodong Xu, Tao Zeng, Tiande Wen

**Affiliations:** 1 Department of Civil Engineering and Smart Cities, College of Engineering, Shantou University, Shantou 515063, China; 2 Special Ceramics Advanced Manufacturing Engineering Technology Research Center of Guangdong Provincial University, Shantou University, Shantou 515063, China; 3 Engineering and Technology Research Centre of Advanced Composite Materials and Structures Advanced Manufacturing, Shantou University, Shantou 515063, China

**Keywords:** thermal transport, thermal radiation, thermal camouflage, metamaterial

## Abstract

Thermal camouflage technologies manipulate heat fluxes to conceal objects from thermographic detection, offering potential solutions for thermal management in high-power-density electronics. Most reported approaches are aimed at scenarios where the target is not a heat source; however, any target with a non-zero temperature emits thermal radiation described by the Stefan–Boltzmann law since the thermal radiation of an object is proportional to the fourth power of its temperature (T^4^). To address this issue, this study proposes a thermal camouflage device that considers the influence of radiative thermal transfer from the target. The underlying principle involves maintaining synchronous heat transfer separately along both the device and background surfaces. Numerical simulation confirms the feasibility of this proposed thermal camouflage strategy. Moreover, by altering some parameters related to the target such as geometry, location, temperature, and surface emissivity, excellent performance can be achieved using this device. This work advances thermal management strategies for high-power electronics and infrared-sensitive systems, with applications in infrared stealth, thermal diagnostics, and energy-efficient heat dissipation.

## 1. Introduction

Metamaterials offer us an approach to realize diverse artificial materials with counterintuitive properties that do not exist naturally. Thanks to the groundbreaking contributions of our predecessors, many significant achievements of metamaterials have been witnessed [1,2,3,4,5,6,7]. In recent years, thermal metamaterials [8,9] possessing extraordinary thermal control properties have made great progress in solving special thermodynamic problems and offer the ability to manipulate the anisotropy of heat flow through structural design. This has led to the creation of thermal devices with various thermal functions, including thermal cloaks and concentrators [10,11,12,13,14,15,16], multiphysics metamaterials [17,18,19], thermal sensors [20,21], thermal harvesters [22], and thermal information devices [23,24].

Among them, thermal camouflage [25] is one of the novel functionalities that can be realized by the thermal metamaterials. With the continuous development of detection technologies, military targets and weapons are facing increasing threats. Stealth technology, as a counter-identification measure, has drawn great attention from all countries. Stealth technology includes optical stealth, radar stealth, and infrared stealth, etc. However, in the detection equipment of various countries at present, infrared detection plays a dominant role. Therefore, the research on thermal stealth technology has received widespread attention from all countries. In the past few years, many thought-provoking design and processing methods to create metamaterials with the functionality of thermal camouflage have been proposed. The strategy of thermal camouflage involves manipulating the temperature distribution surrounding the target to effectively conceal it within its background environment. Precise control over heat flow is achieved through the design of thermal metamaterials using the transformation thermotics method [10,11,12,13,14,15,16,17] or the scattering cancellation method [23]. Many studies [10,11,12,13,14,26] have shown a successful thermal camouflage effect by utilizing the anisotropic thermal conductivity of thermal metamaterials to create equivalent thermal temperature profiles at certain regions. Despite achieving an equivalent exterior temperature profile, the target remains susceptible to detection through infrared (IR) cameras when observed from the normal direction (Z plane). This highlights that relying solely on in-plane heat conduction is insufficient for achieving comprehensive thermal camouflage. Therefore, alternative approaches must be investigated.

Recently, some studies [27,28,29] have achieved camouflage by adjusting out-of-plane heat conduction. Li [24] used two consecutive thermotics transformations to design a conductive structure without a priori knowledge of the background temperature. They further extended the 2D metamaterials-based thermal camouflage device into a 3D version by 3D printing [25]. Zhang [26] maintained the surface temperatures of the device and background plate from point to point through the method of scattering cancellation. They successfully designed various camouflage devices that cover a hidden target against a background plate, ensuring precise correspondence of surface temperatures between the camouflage device and the background plate in the projection domain. As a result, the covered object is effectively concealed, appearing as if it is nonexistent. However, most previous works [27,28] have neglected the thermal characteristics of the target, focusing primarily on scenarios where the hidden target is not a heat source. As any target emits nonzero temperature thermal radiation described by the Stefan–Boltzmann law, the thermal radiation energy is proportional to the fourth power of the temperature, $Q_{r}=\varepsilon A\sigma T^{4}$. In particular, if the target experiences high temperatures, the dissipation of thermal radiation energy from the target will significantly impact the effectiveness of the thermal camouflage device. This will result in weakened or even compromised camouflage performance of the device. Thus, obviously, it is crucial to acknowledge and account for the target’s role as a heat source, ensuring that thermal radiation is taken into account during the design of thermal camouflage devices. Unfortunately, up to now, there have been limited works [29,30] investigating the camouflage performance of devices considering the target as a heat source. They primarily concentrate on the target as a low-temperature heat source while overlooking the significance of the target’s thermal radiation. Due to the absence of a theoretical design method, a high-temperature thermal camouflage device has never been reported.

Therefore, this work proposes a high-temperature thermal camouflage device that considers the influence of the radiative thermal transfer from the target with the aim of addressing the limitation in this field. Theoretically, we precisely deduce the conditions for the realization of thermal camouflage performance. The theoretical principle is based on maintaining the synchronous heat transfer separately along the device and the background. We unexpectedly find that the performance of thermal camouflage can be further optimized by changing the structure of the thermal camouflage device. The results were validated by numerical simulation. Furthermore, the effects of the geometry and location, the temperature, and the surface emissivity of the target on the performance of the thermal camouflage of the device are discussed.

## 2. Theoretical Analysis

**Figure 1:**
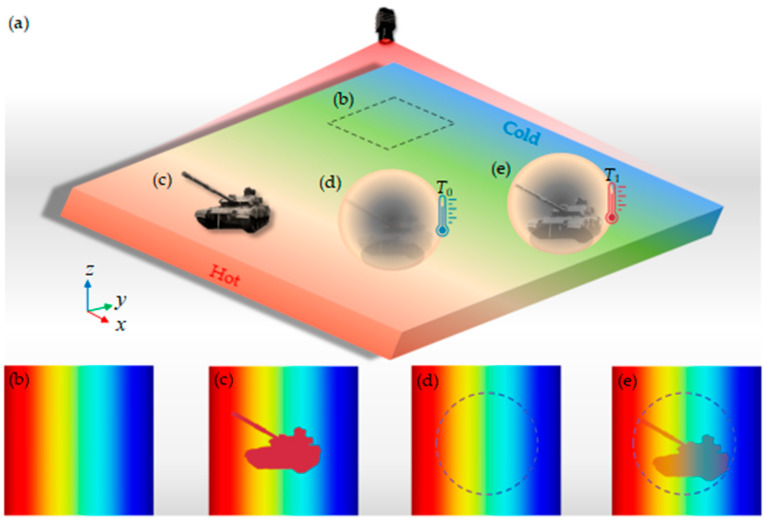
(**a**) The schematic of the different thermal imagery scanned by the infrared thermal imager. (**b**) The thermal image produced by only the bare background plate without any object. (**c**) The target is placed on the basic background plate. (**d**) The characteristic information of the target, which is a non-heat source, cannot be collected from the thermal image when a special device is used to cover the top of the target. (**e**) The heat transferred by the target, which is a heat source, to the external environment inevitably affects the thermal camouflage device and makes the function of thermal camouflage ineffective.

The realistic functional presentation of thermal camouflage can be explained by the illustration shown in Figure 1a–e. There are four different cases shown in Figure 1a. Firstly, as shown in Figure 1b, when the scanning object of the infrared thermal imager is only the bare background plate, all that is obtained is a thermal image of the basic background plate. In addition, when some targets are placed on the basic background plate, the thermal imagery exposes their abnormal information, as shown in Figure 1c. Furthermore, as shown in Figure 1d, when the target is a non-heat source, it exerts no thermal effect on the external environment. By employing a specialized device for target protection, the resulting thermal image closely resembles that of the basic background plate, effectively concealing any pertinent information regarding the target. In contrast, as depicted in Figure 1e, when the target is a heat source, an increase in its temperature leads to heat transfer from the target to its external surroundings, thereby compromising the effectiveness of thermal camouflage and exposing crucial information about the target.

The heat conduction equation that considers heat radiation was adopted to describe the heat transfer process in the radiative thermal camouflage device, which is expressed as follows:

(1) $$\rho_{i}c_{i}\frac{\partial T_{i}}{\partial t}=\nabla \cdot (\kappa_{i}\nabla T_{i})+Q_{r,i}$$

where *T* and *t* denote surface temperature and time; ρ, c, and κ denote the density, specific heat capacity, and thermal conductivity of the object, respectively; and $Q_{r}$ denotes the net radiation heat transfer. The subscript i can denote the target (i=t), background (i=b), and camouflage device (i=d).

According to the Stefan–Boltzmann law, the radiation heat exchange between the objects of the research (i.e., the camouflage device and background) and the target in the closed cavity can be given by

(2) $$Q_{r,i}=F_{t-i}\varepsilon_{t}A_{t}\sigma (T_{t}^{4}-T_{i}^{4})$$

where *F* denotes the configuration factor between different surfaces; ε and $A_{t}$ denote the surface emissivity and the surface area of the target, respectively; and σ is the Stefan–Boltzmann constant equal to 5.669 × 10^−8^ W/(m^2^·K^4^).

**Figure 2:**
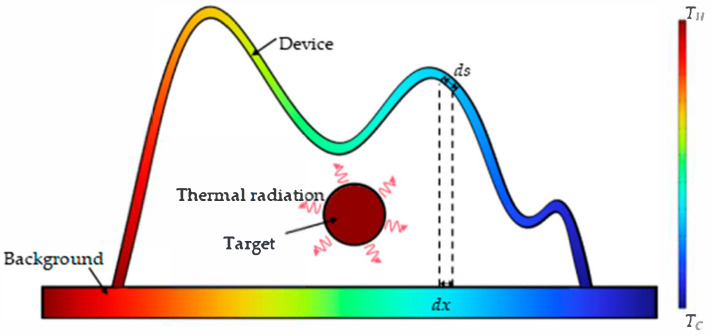
The schematic of the thermal camouflage model.

As shown in Figure 2, the left and right end of the background plate are set to $T_{H}$ and $T_{C}$ ($T_{H}>T_{C}$), respectively, resulting a temperature gradient. There is a target at a high temperature placed above the background radiating thermal energy all around. And the thermal camouflage device is represented by an arbitrary curve covering the background and the target. The theoretical principle used in this work is based on maintaining the synchronous heat transfer separately along the device and the background; when the temperatures of the device and the background are the same, the targets are concealed. For an infinitesimal segment along the background plate, the path integral length of heat conduction is dx, while the counterpart along the camouflage device is the path integral of the device profile, f(x), as $ds=\sqrt{1+f\prime {\left(x\right)}^{2}}dx$. According to the law of conservation of energy, after considering the radiation heat exchange between the target and the objects of the research, the thermal equilibrium equation in the case of two dimensions can be written as


(3) $$\rho_{b}c_{b}\frac{\partial T_{b}}{\partial t}=\frac{\partial }{\partial x}(\kappa_{b}\frac{\partial T_{b}}{\partial x})+F_{t-b}\varepsilon_{t}A_{t}\sigma ({T_{t}}^{4}-{T_{b}}^{4})$$

(4) $$\rho_{d}c_{d}\frac{\partial T_{d}}{\partial t}=\frac{\partial }{\partial s}(\kappa_{d}\frac{\partial T_{d}}{\partial s})+F_{t-d}\varepsilon_{t}A_{t}\sigma ({T_{t}}^{4}-{T_{d}}^{4})$$

Equations (3) and (4) can be further calculated as

(5) $$\frac{\partial T_{b}}{\partial t}=\alpha_{b}\frac{\partial^{2}T_{b}}{\partial x^{2}}+\frac{\partial \alpha_{b}}{\partial x}\frac{\partial T_{b}}{\partial x}+\frac{F_{t-b}\varepsilon_{t}A_{t}\sigma ({T_{t}}^{4}-{T_{b}}^{4})}{\rho_{b}c_{b}}$$

(6) $$\begin{array}{cc} \frac{\partial T_{d}}{\partial t} & =\alpha_{d}\frac{\partial^{2}T_{d}}{\partial s^{2}}+\frac{\partial \alpha_{d}}{\partial s}\frac{\partial T_{d}}{\partial s}+\frac{F_{t-d}\varepsilon_{t}A_{t}\sigma ({T_{t}}^{4}-{T_{d}}^{4})}{\rho_{d}c_{d}}\\ & =\frac{\alpha_{d}}{1+f\prime {\left(x\right)}^{2}}\frac{\partial^{2}T_{d}}{\partial x^{2}}+\frac{1}{1+f\prime {\left(x\right)}^{2}}\frac{\partial \alpha_{d}}{\partial x}\frac{\partial T_{d}}{\partial x}+\frac{F_{t-d}\varepsilon_{t}A_{t}\sigma ({T_{t}}^{4}-{T_{d}}^{4})}{\rho_{d}c_{d}} \end{array}$$

where α denotes the thermal diffusion coefficient, as $\alpha =\frac{\kappa }{\rho c}$.

It can be inferred from Equations (5) and (6) that the requirements $T_{d}(x,t)\equiv T_{b}(x,t)$, when x changes in the range of $\left[a,b\right]$ to be satisfied can only be achieved if the following Equations (7)–(9) are satisfied simultaneously.


(7) $$\alpha_{b}=\frac{\alpha_{d}}{1+f\prime {\left(x\right)}^{2}}$$

(8) $$\frac{\partial \alpha_{b}}{\partial x}=\frac{1}{1+f\prime {\left(x\right)}^{2}}\frac{\partial \alpha_{d}}{\partial x}$$

(9) $$\frac{F_{t-b}}{\rho_{b}c_{b}}=\frac{F_{t-d}}{\rho_{d}c_{d}}$$

Therefore, substituting Equation (7) into Equation (8) yields the following equation:

(10) $$\frac{2f\prime (x)f\prime \prime (x)}{{\left(1+f\prime {\left(x\right)}^{2}\right)}^{2}}\alpha_{d}=0$$

Due to $\alpha_{d}\neq 0$, the solution of Equation (10) is valid only when f′(x)f″(x)=0. When f′(x)=0, the thermal camouflage device is completely attached to the background plate, which does not conform to the theoretical model. Hence, the reasonable solution of Equation (10) is f″(x)=0, which yields the following equation:

(11) f′(x)=C=tanθ

where *C* denotes a constant, and θ denotes the angle between the thermal camouflage device and the background plate. Substituting Equation (11) into Equation (7) yields the following:

(12) $$\frac{\alpha_{d}}{\alpha_{b}}=1+\tan^{2}\theta$$

Equation (12) can further become the following equation.


(13) $$\frac{\alpha_{d}}{\alpha_{b}}=\frac{\frac{\kappa_{d}}{\rho_{d}c_{d}}}{\frac{\kappa_{b}}{\rho_{b}c_{b}}}=\frac{\kappa_{d}}{\kappa_{b}}\frac{\rho_{b}c_{b}}{\rho_{d}c_{d}}=1+\tan^{2}\theta$$

Hence, substituting Equation (9) into Equation (13) can further yield the following:

(14) $$\frac{\kappa_{d}}{\kappa_{b}}\frac{\rho_{b}c_{b}}{\rho_{d}c_{d}}=\frac{\kappa_{d}}{\kappa_{b}}\frac{F_{t-b}}{F_{t-d}}=1+\tan^{2}\theta$$

Namely,


(15) $$\frac{\kappa_{d}}{\kappa_{b}}=(1+\tan^{2}\theta )\frac{F_{t-d}}{F_{t-b}}$$

In this paper, the configuration factor is a parameter that is only related to the geometric structure. Thus, Equation (15) shows that as long as the thermal conductivity of the device and background and the configuration factor can be determined, we can calculate the angle θ from Equation (15) to achieve the thermal camouflage that we expect. Since the camouflage device has to contact the background plate again to form an enclosed interior space, we can use the piecewise linear function with an angle θ to the *X*-axis to describe the profile of the device. Obviously, the resulting device is an isosceles triangle structure. So now the question is how to calculate the configuration factor.

**Figure 3:**
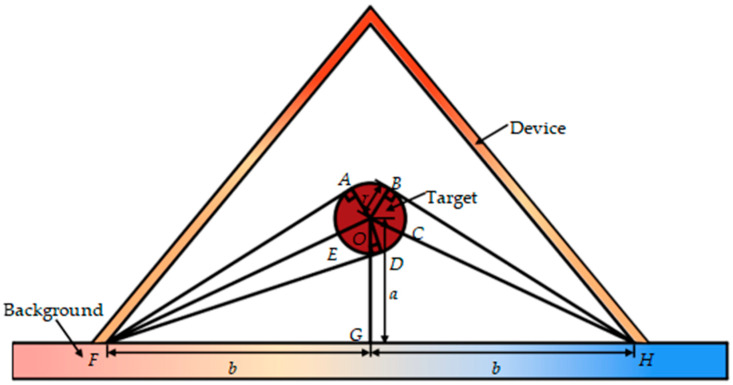
The schematic of the geometric relationships among the background, the device, and the target.

The schematic of the geometric relationships among the background, the device, and the target are shown in Figure 3. Let ∠FOG=∠HOG=α and ∠FOA=∠FOD=∠HOB=β. According to Hottel’s cross-string method, the configuration factor between the background and the target can be written as

(16) $$F_{t-b}=\frac{2\left(\bar{DF}+\overset{⌢}{DB}\right)-2\bar{BH}}{2\overset{⌢}{ADB}}=\frac{\bar{DF}+\overset{⌢}{DB}-\bar{BH}}{\overset{⌢}{ADB}}$$

Further, note that ΔODF≅ΔOBH; thus,


(17) $$\bar{DF}=\bar{BH}$$

(18) ∠FOD=∠BOH

Substituting Equation (17) into Equation (16) results in

(19) $$F_{t-b}=\frac{\overset{⌢}{DB}}{\overset{⌢}{ADB}}$$

Note that $∠FOH=∠BOD=2\alpha =2\arctan\left(\frac{b}{a}\right)$; thus,

(20) $$\overset{⌢}{DB}=2r\arctan\left(\frac{b}{a}\right)$$

In addition, according to the Law of Sines,

(21) $$\sin\beta =\frac{\bar{FD}}{\bar{FO}}=\frac{\sqrt{a^{2}+b^{2}-r^{2}}}{\sqrt{a^{2}+b^{2}}}$$

Therefore, we can derive

(22) $$\overset{⌢}{ADB}=r\left(2\alpha +2\beta \right)=2r\left(\arctan\left(\frac{b}{a}\right)+\arcsin\left(\frac{\sqrt{a^{2}+b^{2}-r^{2}}}{\sqrt{a^{2}+b^{2}}}\right)\right)$$

Thus, substituting Equations (20) and (22) into Equation (19) results in

(23) $$F_{t-b}=\frac{\overset{⌢}{DB}}{\overset{⌢}{ADB}}=\frac{\arctan\left(\frac{b}{a}\right)}{\arctan\left(\frac{b}{a}\right)+\arcsin\left(\frac{\sqrt{a^{2}+b^{2}-r^{2}}}{\sqrt{a^{2}+b^{2}}}\right)}$$

Furthermore, for a closed cavity,


(24) $$F_{t-d}+F_{t-b}=1$$

Consequently, substituting Equations (23) and (24) into Equation (15) results in


(25) $$\frac{\kappa_{d}}{\kappa_{b}}=(1+\tan^{2}\theta )\frac{F_{t-d}}{F_{t-b}}=(1+\tan^{2}\theta )\frac{\arcsin\left(\frac{\sqrt{a^{2}+b^{2}-r^{2}}}{\sqrt{a^{2}+b^{2}}}\right)}{\arctan\left(\frac{b}{a}\right)}$$

Therefore, with Equation (25), once we know the material parameters and the geometric relationships of the three objects, we can design the thermal camouflage device with the triangle shapes at the inclination angle θ.

## 3. Results and Discussion

**Figure 4:**
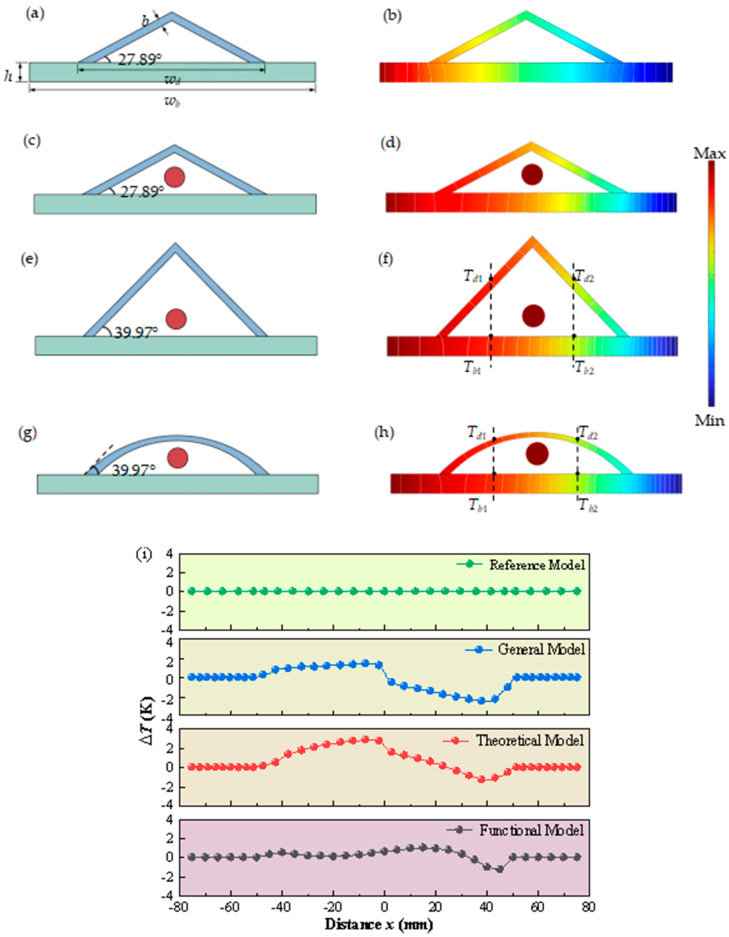

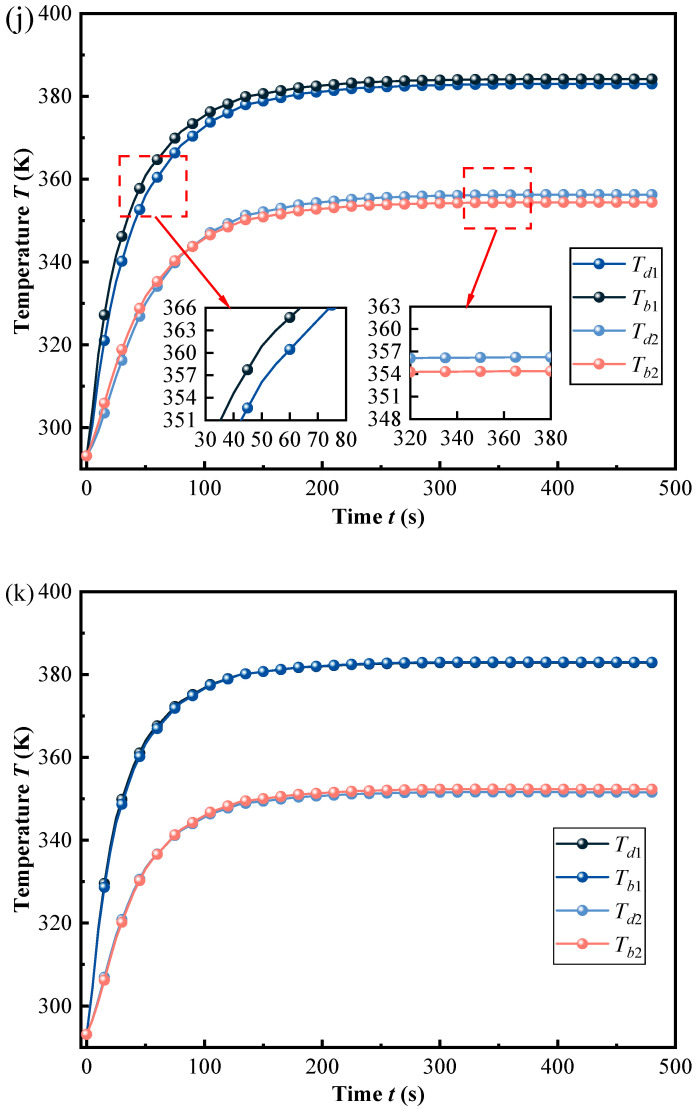
The diagram of (**a**) the reference model, (**c**) the general model, (**e**) the theoretical model, and (**g**) the functional model and the temperature distribution of (**b**) the reference model, (**d**) the general model, (**f**) the theoretical model, and (**h**) the functional model. The white lines are isothermal lines. (**i**) The temperature deviation between the surface temperatures of the device and the background ΔT(x) along the position distribution for each model. The transient temperature field comparison of the certain points between the background and the device for (**j**) the theoretical model and (**k**) the functional model.

In order to verify the validity of the effectiveness of the thermal camouflage device, which considers the influence of the radiative heat transfer of the target, the reference model, the general model, and the theoretical model were established. As shown in Figure 4a, the reference model consists of an isosceles triangular camouflage device and a background plate, with an inclination angle of θ = 27.89° proposed by Zhang [26], disregarding the thermal properties of the camouflaged targets. The reference model was used as the control group to verify the thermal camouflage effect when the target’s thermal radiation was not taken into account. To investigate whether there is an impact on the thermal camouflage device from a target, we established a general model as shown in Figure 4c. The general model resembles the reference model but incorporates a circular target with a radius of 5 mm positioned at a vertical distance of 10 mm from the background plane. In addition, the theoretical model proposed in this paper is shown in Figure 4e, which takes into account the thermal properties of the camouflaged target. It also features an isosceles triangular camouflage device but with an inclination angle of θ = 39.97°. The inclination angle of 39.97° for the theoretical model was recalculated based on our consideration of the characteristics of the target’s thermal radiation. According to Formula (15), the calculation of the inclination angle takes into account not only the thermal conduction path but also the thermal radiation exchange between the target and the background. In both the general and theoretical models, the temperature and surface emissivity of this target are set to 1500 K and 0.8, respectively. Additionally, all models employ copper for constructing the thermal camouflage device, while aluminum alloy 6063 is used for the background material. The material parameters used in the calculation are shown in Table 1. As shown in Figure 4a, the dimensions of the background are set to *w_b_* = 150 mm, *h* = 10 mm, and w*_d_* = 100 mm, and the thickness of the device is *b* = 4 mm.

Subsequently, we performed numerical simulation with the commercial software COMSOL Multiphysics 6.0 to validate the performance of the models. We set constant temperatures on the left and right side to *T_L_* = 400 K and *T_R_* = 293.15 K, respectively. The remaining outer boundaries of the system were considered insulated, while the ambient temperature remained at 293.15 K. Taking the isosceles triangle model as an example, this study assessed whether the mesh density could produce mesh-independent results through temperature evaluation. The main steps included selecting five different mesh densities for the model, balancing numerical accuracy and computational efficiency while avoiding overly dense meshes that would lead to unnecessary computational time. The five mesh densities were categorized as extremely fine, extra fine, fine, normal, and coarse, with the total number of elements being approximately 58,988, 21,505, 5444, 3894, and 1669, respectively. The temperatures calculated using the normal and coarse mesh densities showed significant differences from the theoretical predictions. However, when using the fine and extra fine mesh densities, the calculated temperatures were very close to the theoretical predictions. Moreover, there was almost no difference in the results obtained using the extra fine and extremely fine meshes, indicating that mesh-independent results were achieved when the number of elements was around 21,505. Therefore, this mesh density and the corresponding element type were adopted for the subsequent numerical analysis, and the temperature distribution and evolution were subsequently obtained. The temperature distributions of the reference model, the general model, and the theoretical model are shown in Figure 4b,d,f, respectively. The performance of the thermal camouflage is quantified using the temperature deviation between the surface temperatures of the device and the background ΔT(x) along the position distribution [$\Delta T(x)=T_{b}(x)-T_{d}(x)$]. As shown in Figure 4i, the temperature deviation ΔT of the reference model is identically equal to zero, meaning the surface temperatures of the device and the background are exactly the same. The reference model is the control group for this simulation. Compared with the reference model, under the effect of the thermal radiation heat transfer of the target, the diagram of the temperature deviation ΔT of the general model significantly changes, representing significant deviations and fluctuations. The sharp change in ΔT clearly exposes the presence and contour of the target; this means that the camouflage performance of the thermal camouflage device by the general model becomes weakened or even eliminated. Furthermore, considering the influence of radiative thermal transfer from the target in the theoretical model, it is evident that the temperature deviation ΔT is effectively mitigated within the anterior section of the device.

This abrupt jump in ΔT observed within the posterior section is greatly suppressed by the modified theoretical model (named the functional model), which incorporates tangent arcs on both sides of the original theoretical model (the triangle structure). The functional model, depicted as an arc structure in Figure 4g, exhibits the temperature distribution shown in Figure 4h. As shown in Figure 4i, the temperature deviation ΔT of the functional model does not produce a sharp change at the coordinate x = 0 but remains closer to zero throughout the whole structure. This variation can be attributed to the influence of heat concentration on heat radiation transfer caused by the acute top angle of the triangular structure, which is eliminated by the smooth surface of the arc structure. The arc-shaped structure, through its smooth geometric shape, avoids the convergence of heat flow at the apex, allowing the heat flow to be more evenly distributed on the arc-shaped surface, thereby preventing the formation of high-temperature areas. The functional model demonstrates a maximum deviation value of 1.2 K, representing its smallest magnitude among all three models.

In addition, as depicted in Figure 4f,h, we extracted and plotted the surface temperature of specific points on both the background and the device over time at two different horizontal locations for the theoretical model (Figure 4j) and the functional model (Figure 4k), respectively. It is worth noting that $T_{d}$ and $T_{b}$ exhibit a similar trend over time, eventually reaching a steady state for both models. However, in the case of the theoretical model, there is a noticeable deviation between the curves of $T_{d}$ and $T_{b}$ over time. Specifically, there is a maximum difference of 6.4 K (2.8 K) between the curves of $T_{d1}$ and $T_{b1}$ ($T_{d2}$ and $T_{b2}$), indicating non-synchronous heat conduction through both the device and the background. Such deviations from synchronous heat transfer can be easily detected using an infrared thermal imager, thereby revealing not only its presence but also its location accurately. In contrast, for the functional model, there is excellent agreement between $T_{d}$ and $T_{b}$ throughout all stages of temperature change, further confirming its exceptional performance in synchronous heat transfer. In summary, except when effectively matching steady-state temperature fields, it can be concluded that the functional model exhibits outstanding performance in terms of synchronous heat transfer over time.

**Figure 5:**
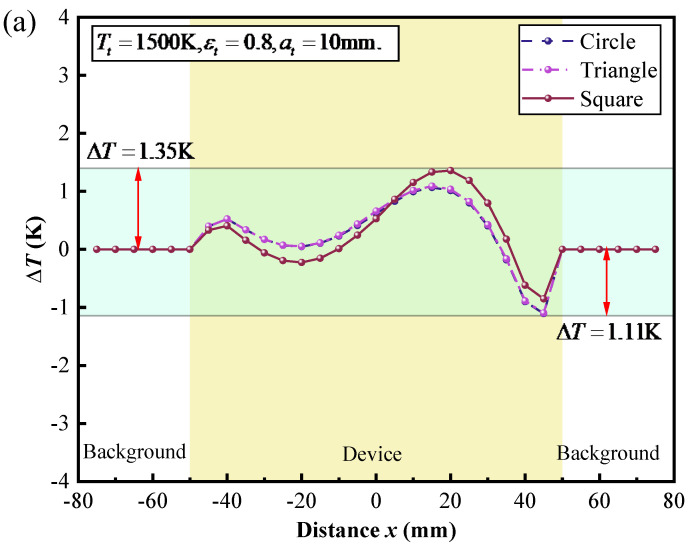

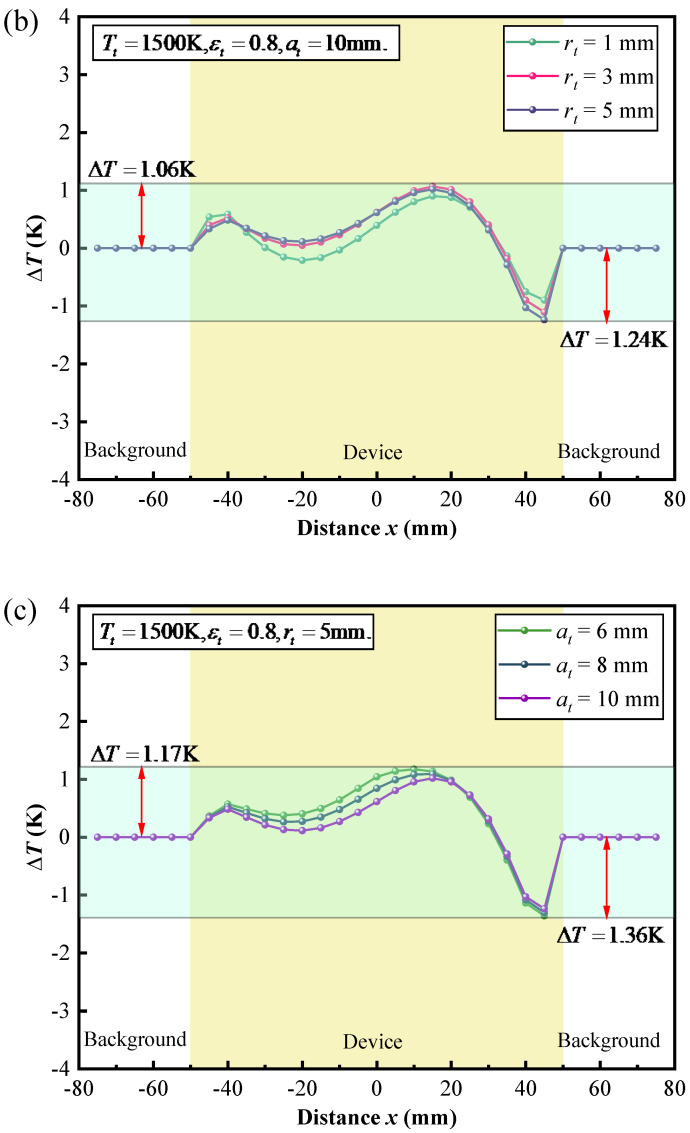

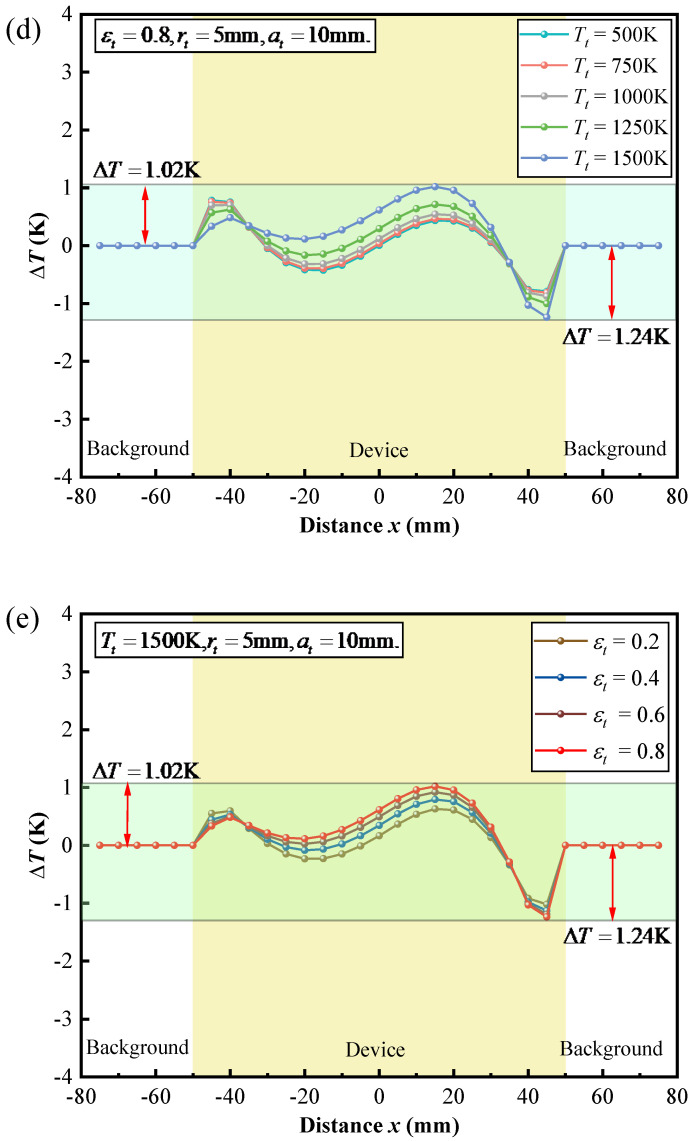
The temperature deviation between the surface temperatures of the device and the background ΔT(x) along the position distribution in the case of (**a**) different shapes, (**b**) different surface areas, (**c**) different locations, (**d**) different temperatures, and (**e**) different surface emissivities of the target.

According to Equation (2), the radiation heat exchange between the camouflage device or background and the target $Q_{r,i}$ is related to the configuration factor between different surfaces F, the surface emissivity ε, the surface area of the target A, and the temperature of the target T. Therefore, in order to further validate the thermal camouflage performance of the functional model, a comprehensive analysis is presented in Figure 5a–e, encompassing various parameters such as the target geometry and location, temperature, and surface emissivity. To simplify the discussion, only one parameter is varied, while the other parameters are kept fixed. In the following discussion, the boundary conditions remain unchanged.

The effect of the geometry and the location of the target on the thermal camouflage performance of the thermal camouflage device was studied, as depicted in Figure 5a–c. Firstly, we systematically varied the shapes of the target, including a circle, triangle, and square, while maintaining a constant surface area. Subsequently, we maintained a circular shape for the target and adjusted its surface area by altering the radii values to 1 mm, 3 mm, and 5 mm. Finally, we employed a circular geometric target with a radius of 5 mm and varied the position of the target within the cavity between the camouflage and background by changing the y-coordinate of the target center by 6 mm, 8 mm, and 10 mm while keeping the x-coordinate constant. By combining Figure 5a–c, it can be found that regardless of the change in the shape, the surface area, or the position of the target, the temperature deviation ΔT always agrees closer to zero.

The effect of the temperature and surface emissivity of the target on the thermal camouflage performance of the thermal camouflage device is illustrated in Figure 5d,e, respectively. As shown in Figure 5d,e, even with significant increases in target temperature and surface emissivity, the temperature deviation ΔT consistently approaches zero. These results demonstrate that the functional model maintains excellent thermal camouflage performance.

## 4. Conclusions

In summary, in stark contrast to the previous thermal camouflage metamaterials, which do not consider the thermal characteristics of the target, we proposed a high-temperature thermal camouflage device that considers the influence of radiative thermal transfer from the target. The theoretical principle of the device is based on simultaneous heat transfer between the device and the background at all times. The performance of the device is confirmed by numerical simulations. Specifically, the functional model (arc-shaped device) achieves near-zero temperature deviation (1.2 K) under steady-state conditions, outperforming the theoretical model (traditional triangular-shaped device). Additionally, the arc-shaped device based on the functional model demonstrates consistent thermal camouflage performance across various target geometries (circle, triangle, and square), vertical positions (6–10 mm), temperatures (500–1500 K), and surface emissivities (0.2–0.8). In the future, by integrating this design with existing frameworks, it may be possible to develop robust thermal camouflage systems suitable for high-temperature environments. In this study, we employed simple triangular and arc-shaped structures for our research. Moving forward, we can explore more complex structures, such as fractal and porous structures, to optimize the design of thermal camouflage devices and achieve better camouflage effects. While this study primarily focused on steady-state thermal camouflage, real-world applications often involve dynamic environmental conditions. Future work can investigate dynamic thermal camouflage technologies and develop devices that can respond in real time to changing conditions. Moreover, this research has the potential to extend its applications to areas such as infrared stealth in optoelectronics, thermal diagnostics for high-power chips, and dynamic heat flux control in integrated circuits.

## Figures and Tables

**Table 1:** Various parameters of the materials used in the device and the background.

	Device	Background
Materials	copper	aluminum alloy 6063
Thermal conductivity (W/(m·K))	398	218
Density (kg/m^3^)	8930	2690
Specific heat capacity (J/(kg·K))	386	900
Infrared emissivity	0.04	0.095

## Data Availability

The datasets used and/or analyzed during the current study are available from the corresponding author on reasonable request.
